# Deep learning and machine learning integration of radiomics and transcriptomics predicts response-adapted radiotherapy outcome and radiosensitivity in resectable locally advanced laryngeal carcinoma

**DOI:** 10.3389/frai.2025.1738174

**Published:** 2026-01-12

**Authors:** Shafat Ujjahan, Abu Shadat M. Noman, Sarah S. Al-Johani, Zakia Shinwari, Ayodele A. Alaiya, Syed S. Islam

**Affiliations:** 1 Department of Radiotherapy, Chattogram Maa O Shishu Hospital & Park View Hospital, Chittagong, Bangladesh; 2 Department of Biochemistry and Molecular Biology, University of Chittagong, Chittagong, Bangladesh; 3 Department of Molecular Oncology, King Faisal Specialist Hospital & Research Centre, Riyadh, Saudi Arabia; 4 Therapeutics & Biomarker Discovery for Clinical Application, King Faisal Specialist Hospital & Research Centre, Riyadh, Saudi Arabia; 5 Institute of Medical Science, Al-Faisal University, Riyadh, Saudi Arabia

**Keywords:** deep learning, head and neck cancer, machine learning, radiomics, radiotherapy, transcriptomics

## Abstract

**Background:**

Radiotherapy (RT) remains a cornerstone treatment for head and neck cancer squamous cell carcinoma. However, therapeutic responses vary considerably among patients due to radiation resistance, which limits long-term survival and contributes to recurrence and disease progression. Developing robust deep learning (DL) and machine learning (ML)-based predictive models is essential to improve response prediction, evaluate treatment outcomes, and identify biomarkers linked to radiosensitization.

**Methods:**

This single-center retrospective study applied DL and ML models to analyze CT scans and RNA-seq gene expression data for prognostic and biomarker discovery purposes. For image analyses, two independent datasets were used. Dataset A includes 1,100 CT scans (pre- and post-treatment) from 476 patients with stage III and IV laryngeal carcinoma treated with response-adapted RT. A convolutional neural network (CNNs) integrated with a recurrent network (RNNs) was used for single-point tumor localization and response prediction. Dataset B, comprising 500 scans from 169 patients treated with radical RT, served as the additional validation cohort. Pre- and post-treatment scans were used to train a DL model, which showed better prediction performance for survival and disease-specific outcomes, including progression and locoregional recurrence. For gene expression-based biomarker analysis, TCGA data (*n* = 231) were examined using glmBoost, support vector machine classifier (SVM), and random forest (RF) algorithms to construct and predict genes associated with radiosensitivity, and the GSE20020 dataset was used to validate the model performance. Proteins and mRNA were used to confirm the signature biomarkers using qRT-PCR and LC–MS mass spectrometry.

**Findings:**

For CT scan image analysis, the DL-model achieved AUCs of 0.792 (*p* = 0.031) at 2-month and 0.832 (*p* < 0.01) at 6-month follow-up. Risk scores significantly correlated with overall survival (HR 1.59, 95% CI 1.34–3.22, *p* = 0.063), progression-free survival (1.39, 95% CI 1.16–2.29, *p* = 0.103). The pathological response in dataset B was likewise significantly predicted by the model. Among 39 differentially expressed genes, ML-model analysis identified 13 candidate genes associated with radiosensitivity on repeated cross-validation with an AUROC of 0.91 in the training set. In the validation dataset, when the models were optimized, the models consistently predicted seven core genes, achieving AUCs ranging from 0.96 to 0.94 to predict the radiosensitivity.

**Interpretation:**

These findings highlight the effectiveness of DL and ML approaches in integrating imaging and transcriptomic data to predict response-adapted RT response and patient outcomes. These automated, and interpretable AI-driven biomarkers hold significant potential for clinical translation. Future research should aim to expand datasets and validate the models in multicenter cohorts for broader applicability.

## Introduction

Cancer of the larynx represents one of the poorest prognoses among head and neck squamous cell carcinoma (HNSCCs). Treatment of this cancer is challenging, as it can profoundly affect speech, swallowing, and breathing (Yamakuni et al., 2023). Given the aggressive nature of this malignancy and the complex anatomy of surrounding functional architecture, both organ preservation and survival remain key treatment goals (Yamakuni et al., 2023; Williamson and Bondje, 2023). Treatment strategies for laryngeal cancer vary by disease stage. In early-stage disease, organ preservation through radiotherapy (RT) or conservative surgery achieves favorable outcomes. Despite the role of surgical resection in achieving maximal local control, laryngeal cancer often carries poor survival, higher risk of salvage surgery, and a long-term complication such as loss of natural voice, tracheotomy dependence, social isolation, swallowing, and breathing impairment (Lagha et al., 2013; Malik et al., 2023).

Response-adaptive approaches have emerged for individualized therapy. Patients demonstrating more than 80% regression after RT or CCRT typically continue non-surgical management, whereas those with <80% regression undergo surgery. This adaptive strategy has improved local control, survival, and organ preservation with an acceptable toxicity profile compared with ICT alone (Lefebvre et al., 2012; Yi et al., 2017). Given these clinical challenges, there is a pressing need to evaluate a response-adapted strategy for laryngeal cancer. The application of artificial intelligence (AI) could be revolutionary in identifying biomarkers linked to radiation response and predicting early tumor responses.

Medical imaging plays a pivotal role in the diagnosis, treatment planning, monitoring progression, and treatment response. Among imaging modalities, computed tomography (CT) remains the most widely used and provides an enormous amount of fine-tuned information acquired by the scanner. Manual interpretation captures only a fraction of this data; therefore, validated computational algorithms are required to extract complex imaging features that may predict therapeutic outcomes. Tracking longitudinal radiographic changes through follow-up scans can offer valuable insights into the dynamics of tumors response dynamics.

Recent advances in AI have transformed cancer imaging and molecular analysis, enabling precise prediction of prognosis, treatment response, and molecular subtypes using CT, histopathology, and transcriptomic data (Cheng et al., 2021; Lan et al., 2024). Deep learning (DL) and machine learning (ML) algorithms have been successfully applied to tumor segmentation, grading, biomarker discovery, and treatment response (Xu et al., 2019). Most DL and ML have been developed to interpret complex spatial patterns of histologic images and features (gene) selection to predict survival and genomic alteration (Rakaee et al., 2025). However, few studies have integrated DL- and ML-based approaches combining radiologic and gene expression data to predict outcomes following response-adapted RT.

In this study, we sought to use AI-driven DL and ML models, specifically convolutional neural networks (CNNs), recurrent neural networks (RNNs), glmBoost, support vector machine (SVM), and random forest (RF) to predict survival and other clinical endpoints of patients with resectable, locally advanced laryngeal carcinoma. We analyzed pre- and post-treatment CT images from patients receiving response-adapted treatment and follow-up CT images following RT alongside RNA-seq data from radiotherapy-resistant and sensitive patient cohorts. Two independent imaging datasets (A and B) with similar diagnoses of stage III and IV laryngeal cancer but treated with different therapy regimens were used for DL model development and validation, while transcriptomic data from TCGA and GSE20020 were used to train and validate ML models predicting radiosensitivity in laryngeal cancer.

## Materials and methods

### Ethics approval and consent to participate

Patient samples were collected from Chattogram Maa O Shishu Hospital, and ParkView Hospital, Chittagong, Bangladesh. The ethics committees at each of the institutes gave their approval to this study. The declaration of Helsinki has been followed in research presented here, and each participant has given their written informed consent to take part in the study.

### DL models for CT scans

#### Study design and data cohort selection

Two independent datasets were used: Dataset A (development cohort) and Dataset B (validation cohort), comprising 645 newly diagnosed patients with resectable stage III and IV laryngeal carcinoma.

##### Dataset A

Dataset A included 476 patients treated with a response-adapted RT strategy using standard-fractionated RT (1.8–2.2 Gy/day, 5 days/week). Initial doses: 70 Gy to gross tumor volume, 60 Gy to tumor-bed area and high-risk clinical target volumes, 50 Gy to prophylactic regions. Then the response-adapted treatment strategy was determined based on the primary tumor response and was evaluated at a dose of 50 Gy. Patients with >80% regression received radical RT or CCRT; others underwent surgery. Partial responders were considered responsive; the rest were nonresponsive. These patients had at least one follow-up CT scan. A total of 1,100 CT scans with an average of 2.31 per patient. In some cases, it was not possible to achieve follow-up scans for some patients. Patients with prior surgery were excluded. The Patients’ cohort was randomly split into a 2:1 ratio, comprising training and development (*n* = 318) for the deep learning model and the other for the test (*n* = 158) cohort, evaluating its performance. The primary endpoint: OS and PFS; secondary: locoregional recurrence and progression with response-adapted RT treatment.

##### Dataset B

Dataset B consisted of 169 patients with similar pathological stages as dataset A, treated with radical RT or CCRT. A total of 500 CT scans were acquired pre- and post-RT. Patients were excluded from this cohort if patients were diagnosed with distant metastasis. The analysis of dataset B was included for further validation with a range of standard care treatment protocols. The primary outcome was pathological response (complete response *vs* residual disease) and locoregional recurrence.

### CT image acquisition and image preprocessing

All CT scans were acquired using Siemens (Germany) according to institutional protocols. Images were obtained pre-contrast and follow-up with varying axial spacing. Slice thickness was 1.0 mm at 120 kVp, with soft tissue reconstruction. Axial, coronal, and sagittal images were obtained 1 min after intravenous administration of 80 mL Omnipaque 350. Image resolution ranged from 0.5 to 0.6 mm with dimensions of 450 × 450 pixels. For both the pre-treatment and the first and second follow-up CT scans following the therapy, the input of the tumor imaging region is defined at the center of the determined seed point. In 3D Slicer 4.8.1, the seed points were manually defined (Xu et al., 2019). The imaging data has to be interpolated to homogeneous resolution in order to provide a steady input for the suggested architectures. This was done since maximum slice thickness was 5 mm and 2D input images were obtained at a non-interpolate slice that was no more than 2 mm distant. Axial slices of 50 × 50 mm^2^, centered on, 5 mm proximal to, and 5 mm distal to the tumor, were input to the model. The model used a ResNet network pretrained on a general image dataset (ImageNet) and the fine-tuned it for CT images (Xu et al., 2019). Three axial slices per time point were used to balance feature representation and computational efficiency. Data augmentation, including flipping, translation, rotation, and small deformation, was applied to all images to reduce overfitting (Krizhevsky et al., 2017). The same augmentation was performed on the pre- and follow-up treatment images, such that the network generates a mapping for the entire input series of images.

### Neural network structure

**Figure 1:**
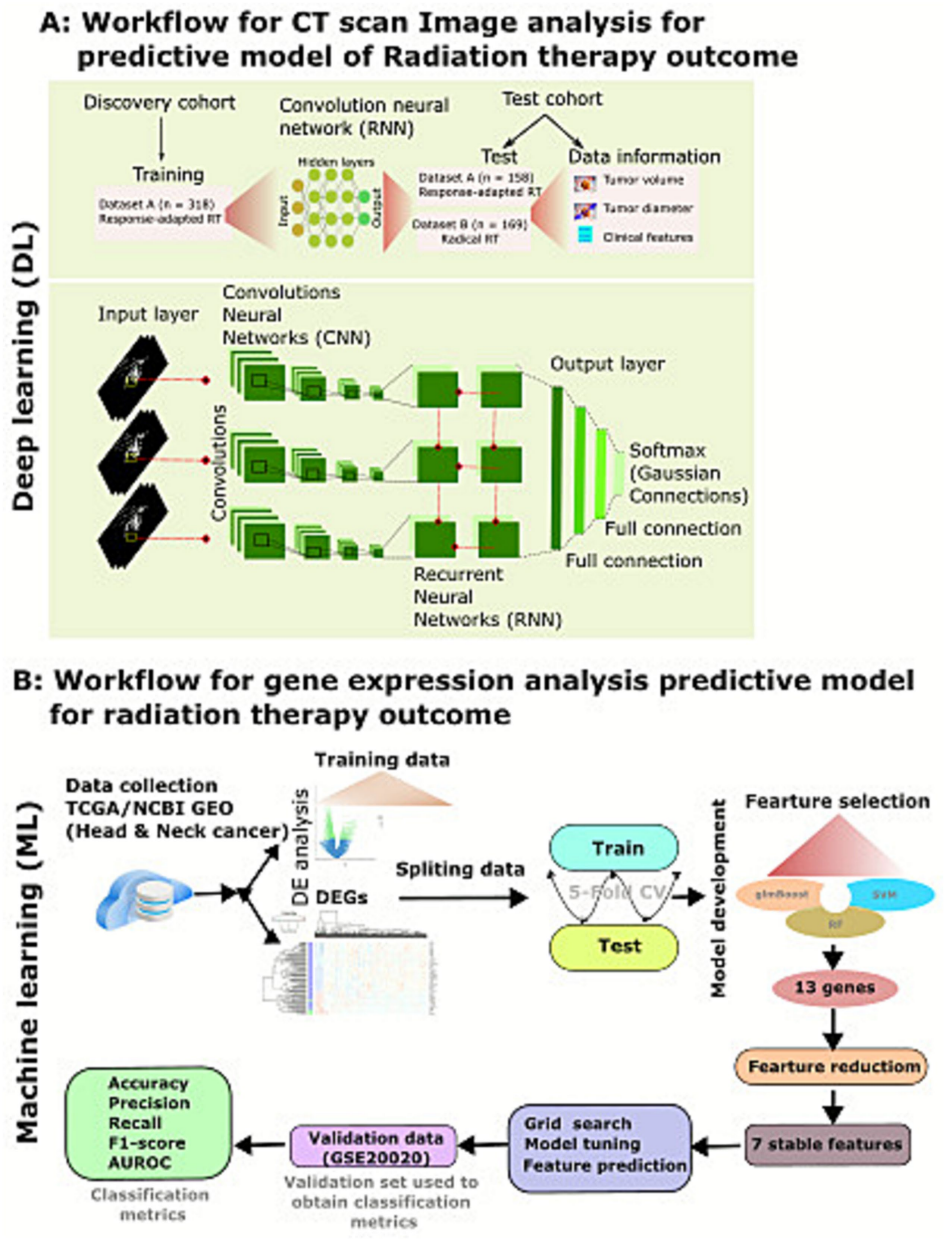
Deep learning analysis model and architecture. **(A)** The model depicts the deep learning model of two datasets. Dataset A consists of 476 patients treated with a response-adapted RT treatment strategy. Dataset A was used for training and fine-tuning the convolutional neural network combined with the recurrent neural network (RNN) for the prediction of survival. The test data set from dataset A was used to assess the performance and compared with the performance of radiographic and clinical features. Dataset B included patients treated with radical radiotherapy. This cohort was used as an additional set to predict pathological response, and the model predictions were compared to changes in tumor volume. The neural architecture includes a convolutional neural network (CNN) merged with a recurrent neural network (RNN) and was trained on baseline and follow-up scans. The input axial slices were 30 × 30 mm^2^ centered on 5 mm proximal to and 5 mm distal to the selected input point. Deep learning networks are trained on natural RGB images and thus require three slices per input. The outputs of each CNN model are input into the RNN, with a gated recurrent unit (GRU) for time-varying inputs. Masking was performed on certain inputs of the CNN scans that the recurrent network takes into account missed scans. The final softmax layer provides the prediction. **(B)** Machine learning framework to identify signature genes from differentially expressed genes dataset obtained from TCGA. Three feature selection algorithms glmBoost, supporting vector machine (SVM), and random forest (RF) were used to identify signature genes. Subsequently, feature elimination methods and tested model performance using GSE20020 dataset to predict patients’ radiotherapy outcome.

The model was implemented in Python, using Keras with a TensorFlow backend (Python 3.0, Keras 2.0.8, TensorFlow 1.3.0). The proposed network structure has a base ResNet convolutional neural network (CNN) trained on the database containing over 14 million neural images. One CNN was defined for each time point input, such that an input with scans at three time points would involve input into three CNNs. The output of the pretrained network model was then input into recurrent layers with a gated recurrent unit (GRU), which takes the time domain into account. The output of the pretrained network was masked to skip the time points. Averaging the fully connected layers is then applied after GRU with batch normalization and dropout after each fully connected layer to prevent overfitting (Ioffe and Szegedy, 2015; Srivastava et al., 2014). The final softmax layer allows for a binary classification output (Figure 1A). To test the model without the input of follow-up scans, the pre-treatment images alone were input into the proposed model, with the recurrent and average pooling layers replaced by fully connected layers, as there was only one input time point. For model training and transfer learning procedures, dataset A was divided into training and testing subsets using 2:1 ratio. Model training was conducted using Monte Carlo cross-validation, employing five randomized stratidfied splits across a cohort of 318 patients. Each split was trained for up to 200 epochs with class-weight balancing to account for outcome imbalance. Model performance was evaluated using an independent cohort comprising 158 patients who were not included in any part of training or hyperparameter tuning. This validation set served as to assess models’ generalizability and robustness to previously unseen data. For all experiments, pre-treatment images were used as input to the proposed model. Within the transfer-learning network, the original recurrent and average-pooling components of the architecture were replaced with a fully connected layers to better capture high-level imaging features relevant to the classification task.

### Machine learning (ML) model and transcriptome data

#### Training data set: TCGA RNA-seq expression data, prediction, and patients’ prognosis evaluation

TCGA (The Cancer Genome Atlas) RNA-seq head and neck cancer count data (downloaded on May 2025) were retrieved via the TCGAbiolinks package in R. After preprocessing, 447 patients had complete clinical data; among these, 235 had laryngeal primary tumors. Genes with counts <5 were excluded, and the remaining data were normalized using the Limma (v3.64.3) R package. Differential expression (DE) analysis between radiation-sensitive and -resistant groups was performed using DESeq2.

### Gene set enrichment analysis (GSEA) and enrichment analysis

GO and KEGG enrichment analyses were conducted using Python’s GSEApy package, GO Biological Process (GO-BP-2025), and KEGG-Human-2025 databases were used, with significance defined as adjusted *p* < 1e-3.

### Application of machine learning

#### Machine learning model construction

In our training data set, gene expression raw data were obtained from TCGA, and differential analysis was performed, and the differentially expressed genes (DGEs) to develop a binary classification model using machine learning (ML) to predict radiotherapy outcomes in head and neck cancer patients. The entire ML analysis pipeline is summarized in Figure 1B. Due to the asymmetrical nature of the gene expression data in the training dataset, we applied a 5-fold stratified cross-validation process (Atreya et al., 2024; Banerjee et al., 2021), randomly partitioning the training dataset into five subsets. We termed this step as 5-fold cross-validation. Then, a feature selection method was applied to genes identified by DEGs to reduce the genes and applied non-linear approaches to identify an optimal set of highly discriminative genes. Our main goal for recursive feature elimination criterion was to discover a limited subset of features to remove redundancy and avoid overfitting. This strategy removes redundant features from the pooled feature set by removing them iteratively and developing a model of the remaining features. To implement the final feature selection method, we employed the feature selection models’ REFCV function. The REFCV function was invoked for each constructed classifier using a 3-fold cross-validation splitting technique and a “ROC-AUC” scoring method using a function parameter.

#### Preferred models

Considering the nature of the training data set and sample size, we choose three popular variable selection models: “glmBoost,” “random forest (RF),” and “support vector machine classifier (SVM).” Both glmBoost and RF are classified as soft classifiers, while SVM is a hard classifier. The gene features selected by each of the models were aggregated into a single input feature set, and the list of DGEs obtained from the training data set was added to this list. We employed a recursive feature elimination process to reduce the genes through feature selection methods. All the models were carried out using a Python-based library, scikit-learn.

#### Evaluation metrics

As the number of resistant and sensitive cases in the training cohort varies significantly (resistant cases 132, and sensitive cases 344), metrics for model evaluation were reported metrics as: accuracy (ratio of all observations correctly predicted by the model), precision (positive predictive values), recall (sensitivity), and F1-score (harmonic mean of precision and recall). In addition, we computed and presented the area under the ROC (receiver operating characteristics) curve (AUC) in evaluating how efficiently the models are separating resistant and sensitive patients.

#### Experimental set-up

To minimize the errors from imbalance treatment outcome in the training dataset, each model was trained with different hyperparameters for tuning via a grid search technique on a subset of training data. We used cross-validation (CV) on the training cohort to select the best hyperparameters from each model. In CV, the data set was split into *K*-folds, with one-fold being left out for model evaluation and the rest used for training. The process is repeated *K*-times, using a different left-out fold each time. Hence, the final evaluation metric for a given model and a set of hyperparameters was computed as the average of the accuracies obtained on the left-out folds at each iteration. The fraction of times a particular gene was chosen out of each iteration was used to rank the genes in descending order for association strength. Genes associated with the outcome of interest in repeated cross-validation analyses were retained and tested for model optimization on the validation cohort.

### Validation dataset

For validation of our training results, we used the GSE20020 ArrayExpress dataset published by Thibodeau et al. (2015) consisted of 19 head and neck cancer patients. This dataset contains 12 samples of complete response (sensitive) and seven samples had radiation treatment failure (resistant). In this dataset, we trained multiple feature sets of the top genes identified through the training dataset to tune the optimal number of features, sampling methods, and classifier combinations, and optimal probability threshold in the validation dataset.

### In-solution protein digestion and mass spectrometry

Crude serum samples from two subject groups of sensitive and resistant patients were analysed using quantitative proteomics. For each group, 100 μg of protein was subjected to in-solution tryptic digestion before LC–MS/MS analysis, as previously described (Alaiya et al., 2021). Samples were heat-denatured at 80 °C for 15 min, and reduction was achieved with 10 mM DTT at 60 °C for 30 min. Samples were alkylated using 50 mM iodoacetamide (IAA) for 30 min at room temperature in the dark. Proteins were digested overnight at 37 °C with sequencing-grade trypsin (Promega, USA) at a 50:1 protein-to-trypsin ratio. After digestion, peptides were diluted with 0.1% formic acid to a final concentration of 1 μg/μL, and 3 μL of each sample was injected for LC–MS/MS analysis. To enable absolute quantification, all samples were spiked with yeast alcohol dehydrogenase (ADH; UniProt ID: P00330) as an internal standard.

### Mass spectrometry platform

The peptide mixture was analysed using nanoAcquity UPLC coupled to a Synapt G2 HDMS instrument with a Trizaic Nano-Flow source (Water, Manchester, UK). Data acquisition was performed using HDMSE (High Definition MSE) mode with the following settings: m/z range 50–2,000 Da, 120 min gradient run time, and ion mobility mode of each sample was analysed in triplicate using MassLynx v4.1 (SCN833).

### Mass spectrometry data analysis

Data processing and protein identification were conducted using Progenesis QI for Proteomics (QIP) v3.0 (Waters/Nonlinear Dynamics, UK). Differentially expressed proteins (DEPs) between sample groups were identified using Statistical filters: ANOVA, *p* ≤ 0.05, and fold change > 1.5, based on/off filtering, i.e., Proteins exclusively detected in one group. The quantitative analysis used ADH (P00330) as the internal reference. Data were further subjected to principal component analysis (PCA) and hierarchical clustering to identify sample group separations.

### qRT-PCR analysis of the patient’s sample

Total RNA was extracted from serum collected before and after the radiotherapy from each patient using TRIzol reagent (Invitrogen, USA). The RNA concentration was measured using Nanodrop 2000 (Thermo Fisher Scientific). cDNA synthesis was performed using Superscript III First-Strand (Invitrogen, USA), and qRT-PCR was performed using the SYBR green PCR master mix (Islam et al., 2024) (Applied Biosystem, USA). The relative levels of mRNA gene expression were calculated using the 2^ΔΔCT^ method. Differences between treatments were evaluated using an unpaired two-tailed Student’s *t*-test. Supplementary Table S1 contains information on the primers used.

### Statistical analysis

All statistical analyses were performed in Python (version 3.12.7) and R (version 4.1.4). Comparisons between two groups were made; continuous variables were analyzed using Student’s *t*-test, while categorical variables were compared using Fisher’s exact test or the chi-squared test. All predictions were evaluated on training tests set for survival and prognostic factors after the response-adapted strategy treatment group. The clinical endpoints included progression and locoregional recurrence as well as overall survival following response-adapted treatment. The analyses were compared to a random forest clinical model with features of stage, gender, age, tumor grade, performance, and tumor size. Survival and prognostic curves were generated using the Kaplan–Meier method between high- and low-mortality risk groups, stratified at the median prediction probability using the log-rank test using the R package ‘Survminer’ (version 0.0.1). Cox regression analyses were performed for both univariate and multivariate models to estimate hazard ratios. Statistical differences between positive and negative survival groups are assessed using the area under the receiver operator characteristics (AUC) and the Wilcoxon rank-sums test. Model performance was assessed using several metrics, including AUC, accuracy (ACC), precision, sensitivity (SEN), specificity/Recall (SPE), F1-score, and the confusion matrix.

An additional test was performed for the second cohort using a 5-year survival model from the RT-only cohort with one time point. Survival predictions were made from a 5-year survival model trained from the response-adapted treatment-only dataset above. The model predictions were used to stratify patients based on survival and tumor response to RT. The groups were assessed using their respective AUC and were tested with the Wilcoxon rank sum test. This was compared to the volume change after RT and the random forest clinical model with the same features used for the response-adapted treatment dataset.

## Results

### Baseline clinical features of the study cohort

A deep learning-based model was evaluated to identify overall survival using 1,600 CT scans from 645 pre- and post-response-adapted RT patients. There was no difference in the age between the two cohorts (age 62.10 [mean SD 8.9] and age 61.5 [mean SD 10.1]). All patients were predominantly diagnosed with stage III and IV (stage IVA and stage IVB, cStage-AJCC 8th edition). Dataset A consisted of a total of 476 patients treated with response-adapted RT that was used as a cohort for testing and training deep learning biomarkers (Table 1). Most patients in this dataset are male (*n* = 374, 79%). The median response-adapted treatment dose was 58 Gy (range 40–70 Gy). The median follow-up period was 42.5 months. The validation cohort (dataset B) served as an additional test set and had 169 patients treated with radical RT. The median radical radiation dose was 70 Gy (range 55–80 Gy), and the median follow-up was 41.5 months. The combined median OS for stage III/Stage IV patients was 12.3 months (95% CI: 8.0–17.8 months). The 1- and 5-year OS were estimated to be 49% (95% CI: 47–53%) and 28% (95% CI: 21–43%), respectively (Supplementary Figure S1A). Furthermore, median disease-free survival was 17.2 months (95% CI 11.6–30.1 months) (Supplementary Figure S1B).

**Table 1:** Baseline patient characteristics of the training and testing cohort of dataset A (response-adapted radiation treatment group).

Variables	*N* = 476^1^	Training cohort *N* = 318^1^	Testing cohort *N* = 158^1^	*p*-value^2^	*q*-value^3^
Sex					
Male	374 (79%)	247 (78%)	127 (80%)	0.5	0.7
Female	102 (21%)	71 (22%)	31 (20%)		
Age, median (range), y	62.12 (9.8)	61.0 (8.9)	61.5 (10.1)	0.2	0.4
Subsites					
Epiglottis	131 (28%)	98 (31%)	3 (2%)	0.012	0.027
Glottis	76 (16%)	49 (15%)	27 (17%)		
Subglottis	80 (17%)	61 (19%)	19 (12%)		
Supraglottis	72 (15%)	33 (10%)	39 (25%)		
Vocal cord	117 (25%)	77 (24%)	70 (44%)		
CCI^4^-score					
0	194 (41%)	140 (44%)	54 (34%)	0.8	0.8
1	171 (36%)	124 (39%)	47 (30%)		
2	70 (15%)	37 (12%)	33 (21%)		
>3	41 (9%)	17 (5%)	24 (15%)		
Clinical T-stage					
T1	22 (5%)	13 (4%)	9 (6%)	0.7	0.8
T2	43 (9%)	21 (7%)	22 (14%)		
T3	197 (41%)	133 (42%)	64 (40%)		
T4	214 (45%)	151 (48%)	63 (40%)		
Clinical N-stage					
N0	17 (4%)	11 (3%)	6 (4%)	<0.0001	<0.001
N1	19 (4%)	9 (3%)	10 (6%)		
N2	310 (65%)	201 (63%)	109 (69%)		
N3	130 (27%)	97 (30%)	33 (21%)		
Clinical stage					
III	64 (13%)	55 (17%)	9 (6%)	0.8	0.8
IVA	329 (69%)	198 (62%)	131 (83%)		
IVB	83 (17%)	65 (20%)	18 (11%)		
Concurrent chemotherapy	146 (31%)	129 (41%)	17 (11%)	0.007	0.019
Radiation techniques					
3DCRT^5^	18 (4%)	15 (5%)	3 (2%)	0.006	0.019
IMRT^6^	450 (95%)	298 (94%)	152 (96%)		
VMAT^7^	8 (2%)	5 (2%)	3 (2%)		
ECOG^8^					
0	14 (3%)	11 (3%)	3 (2%)	<0.001	<0.001
1	460 (96%)	306 (96%)	154 (97%)		
>2	2 (1%)	1 (1%)	1 (1%)		
Pretreatment evaluation					
Laryngeal preservation	142 (30%)	109 (34%)	33 (21%)	0.5	0.7
Total laryngectomy	334 (70%)	209 (66%)	125 (79%)		

^1^Mean (SD); *n* (%); ^2^Wilcoxon rank sum test: Pearson’s Chi-squared test: Fishers exact test; ^3^False discovery rate correction for multiple testing; ^4^Charlson Comorbidity Index; ^5^Definitive Chemotherapy; ^6^Intensity Modulated Radiation Therapy; ^7^Volumetric Modulated Arc Therapy; ^8^Eastern Cooperative Oncology Groups.

Median (range) was reported for continuous variables and counts (percentage) for categorical variables.

### Tumor volume

**Figure 2:**
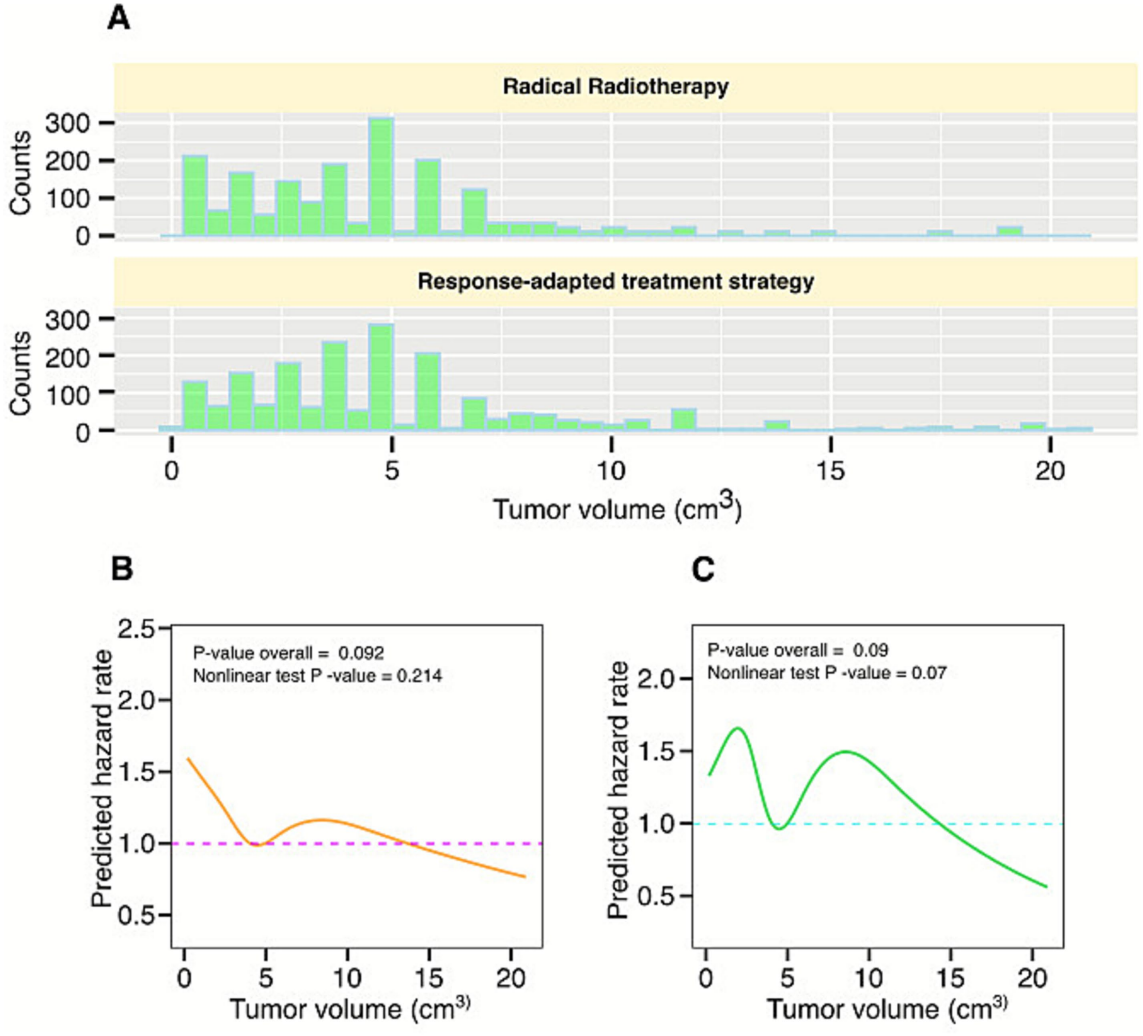
**(A)** The distribution of tumor volume of all patients in dataset A. **(B,C)** Restricted cubic spline analysis showing associations between tumor volume and overall survival and progression-free survival.

The tumor volume was measured, and the mean tumor volume was 4.98 (3.32) cm^3^, with median, minimum, and maximum values found as 4.06 cm^3^, 0.22 cm^3^, and 20.87 cm^3^, respectively. The distribution of tumor volume for the response-adapted RT strategy and radical radiotherapy (RRT) is shown in Figure 2A. Restricted cubic splines (RCS) models based on the Cox proportional hazard model for the entire dataset A were analyzed to visualize the impact of tumor size on prognosis. Results obtained from the RCS model highlighted that the linearity assumption for both OS (*p* = 0.21) and DFS (*p* = 0.07) could not be rejected (Figures 2B,C).

### Deep learning-based prediction of prognostic biomarker and model performance

**Figure 3:**
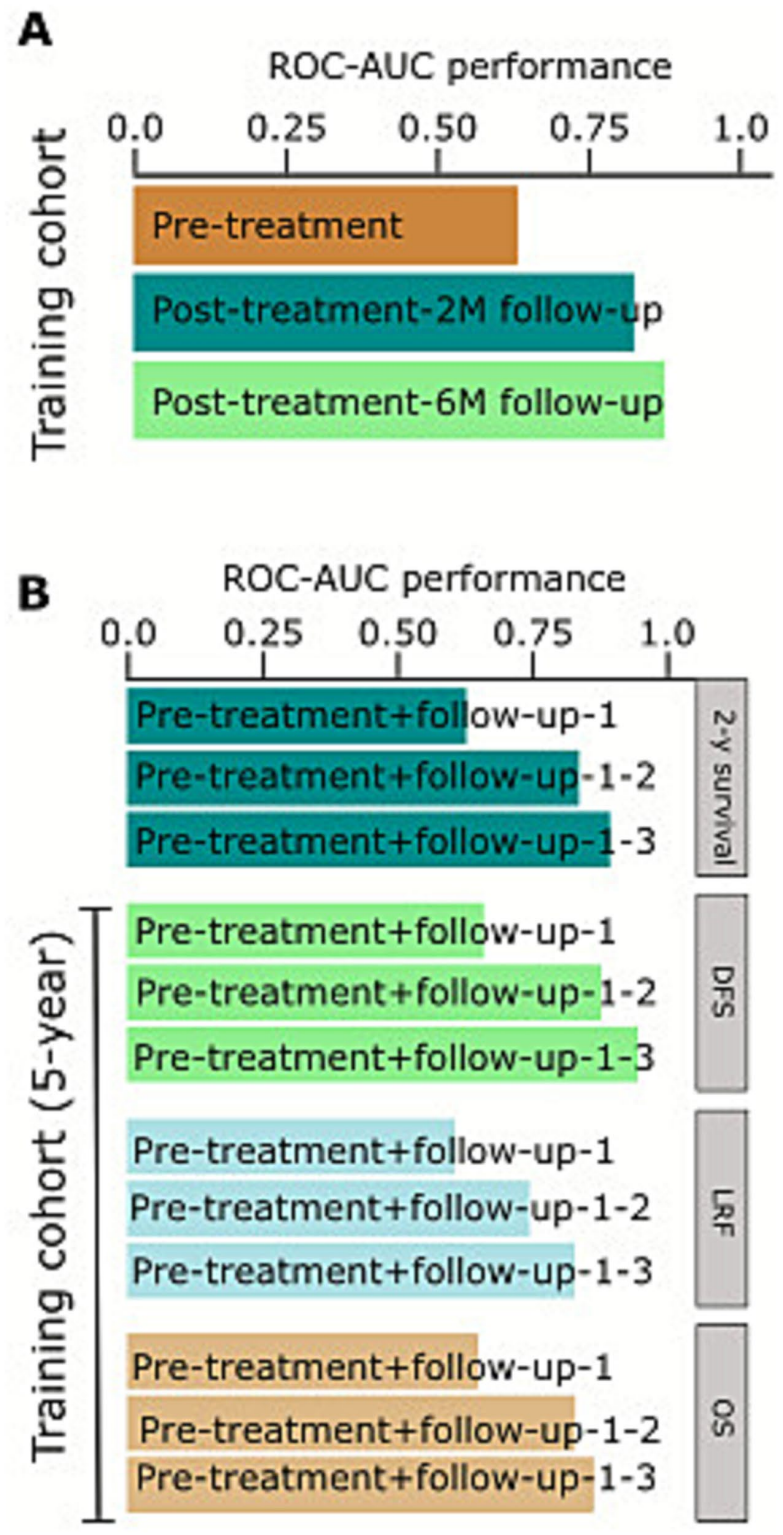
Performance of deep learning biomarkers with the increase in the number of treatment time points. **(A,B)** AUC values were evaluated on an independent test set from the deep learning models for 5-year overall survival (OS), progression-free survival (PFS), and locoregional recurrence-free survival (LRF). Values were significantly separated with Wilcoxon’s rank sum test in the response-adapted RT treatment strategy (dataset A). The response-adapted RT treatment strategy cohort with the input of the pre-treatment scans along with the addition of 1- and 2-follow-up scans at 2- and 6-month follow-up.

The discovery component of dataset A was used for training the model in order to generate deep learning (DL)-based biomarkers for survival, progression-free survival, and locoregional recurrence. The workflow for the prediction is presented in Figure 1A. The discovery cohort was randomly split into a 2:1 ratio for training and development (*n* = 318) of the deep learning model and the remaining for testing and evaluating the performance (*n* = 158). The baseline model with only pretreatment scans showed low performance for predicting 5-year overall survival (AUC 0.60, 95% CI 0.52–0.69; *p* = 0.17, Wilcoxon’s test; Figure 3A). The pretreatment scans achieved low performance, which was lower than the post-treatment 2- and 6-month scans (Figure 3A). The DL model demonstrated strong predictive performance for predicting 5-year overall survival, achieving AUCs of 0.792 (95% CI 0.790–0.817; *p* = 0.031) for the 2-month follow-up scans, while after the 6-month follow-up scans, the AUC increased to 0.832 (95% CI 0.830–0.839; *p* = 0.01; Figures 3A, Supplementary Figure S2). The DL performance model was assessed on other clinical characteristics, i.e., 2-year overall survival and 5-year survival, progression, and locoregional recurrence-free survival (LRF). The model demonstrated comparable performance; however, there was no significant prediction of survival at 2 years (AUC = 0.671) or 5 years (AUC = 0.746, *p* = 0.436; Figure 3B) or treatment response (Supplementary Table S2).

### Deep learning model and response-adapted treatment strategy for clinical endpoints

**Figure 4:**
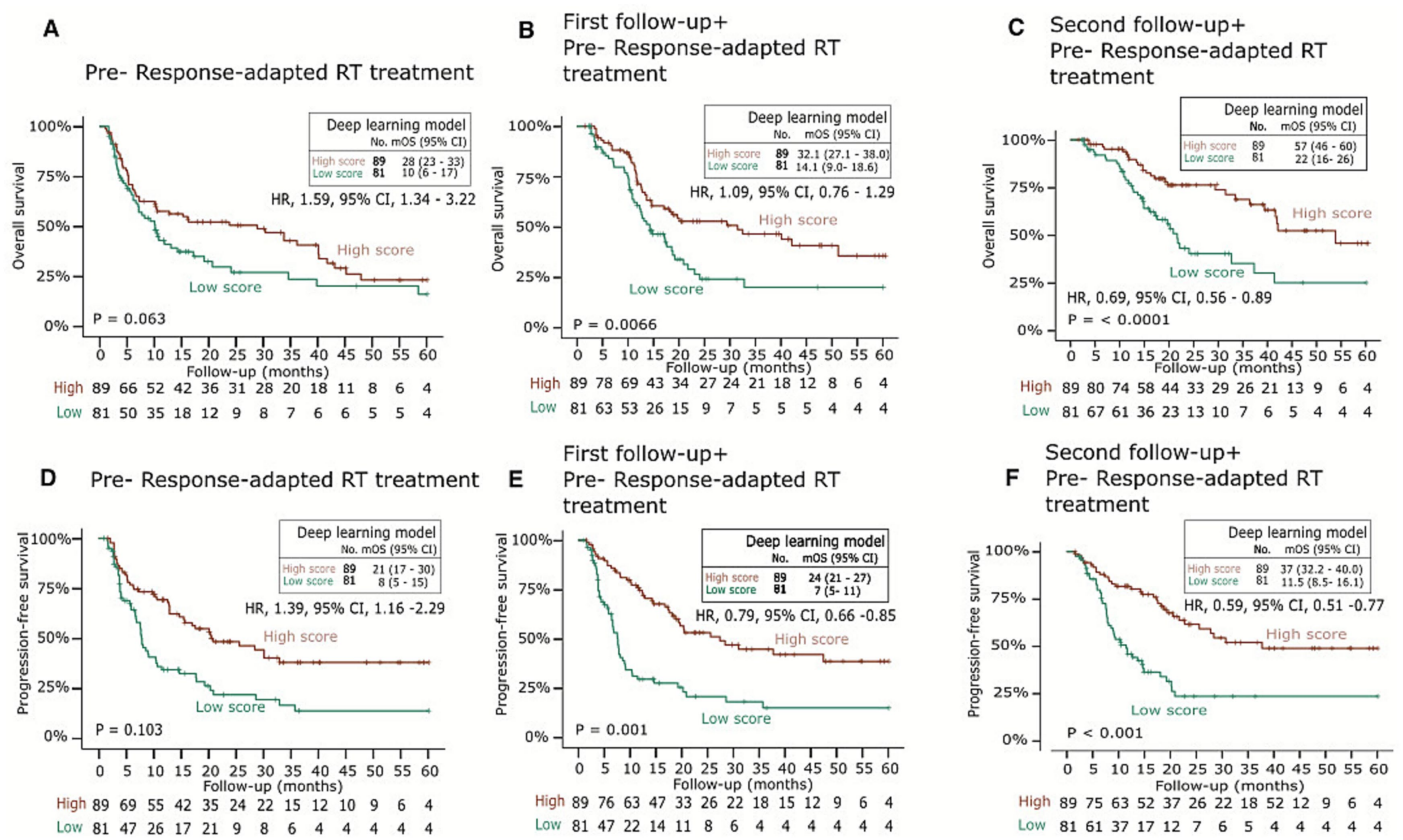
**(A–F)** Performance of DL biomarkers on validation dataset. The DL models were evaluated on an independent test set for performance. The 5-year overall survival Kaplan–Meier curves were generated with median stratification of the low- and high-mortality risk groups with follow-up data. Log rank *p* < 0.05 for >1 follow-up.

The median (IQR) follow-up time for the training cohort was 55.8 months (32.6–60.0) and 38.4 months (32.8–49.1) for the testing cohort. The survival analyses were performed with Kaplan–Meier estimates for high- and low-mortality risk groups based on median stratification of patients’ prediction scores. The model yielded a significant difference between the high and low score groups among 2 (*p* = 0.006, log-rank test) and 3 (*p* < 0.001, log-rank test) follow-up scans. Patients with higher scores (> median) had significantly longer overall survival and progression-free survival in 2 and 3 follow-up scans (Figures 4A–F). Comparable results were found for the following predictions with their respective hazard ratios. The hazard ratio for five-year overall survival was 1.59 (95% CI 1.34–3.22, *p* = 0.063); for progression-free survival, it was 1.39 (95% CI 1.16–2.29, *p* = 0.103); and for locoregional recurrence-free survival, it was 1.87 (95% CI 1.11–5.12, *p* = 0.01), each with significant differences at two follow-up time point scans.

### Predicting pathologic response with the independent validation dataset

To confirm the results from the training set (dataset A), we used additional validation data to determine the relationship between image analysis and pathological response in patients who were treated with radical radiotherapy (primary RT). The pre-RRT and post-RRT scans were given as input to the neural network model trained on dataset A. First, for survival prediction analysis, the model was tested on Dataset B. To match the number of input time points, the 5-year survival model with pre-treatment and first follow-up at 2 months was used. Interestingly, the model significantly predicted progression-free survival and local regional recurrence (Supplementary Table S3). From the results, it appears that the model accurately predicted all clinical endpoints in dataset B.

In predicting the RT response, the predictive network was used to categorize the pathological response. In the validation dataset (*n* = 169), the model demonstrated comparable performance, with an F1-score of 0.67, recall of 0.79, and precision of 0.66. For dataset A of the test set, the model correctly predicted non-responder status for 37 of 45 patients (23%) and responder status for 79 of 113 patients (50%), reaching an overall accuracy of 73%. In the additional validation cohort of dataset B, the model accurately predicted 41 of 63 non-responders (24%) and 64 of 106 responders (40%), achieving a total accuracy of 64%.

**Figure 5:**
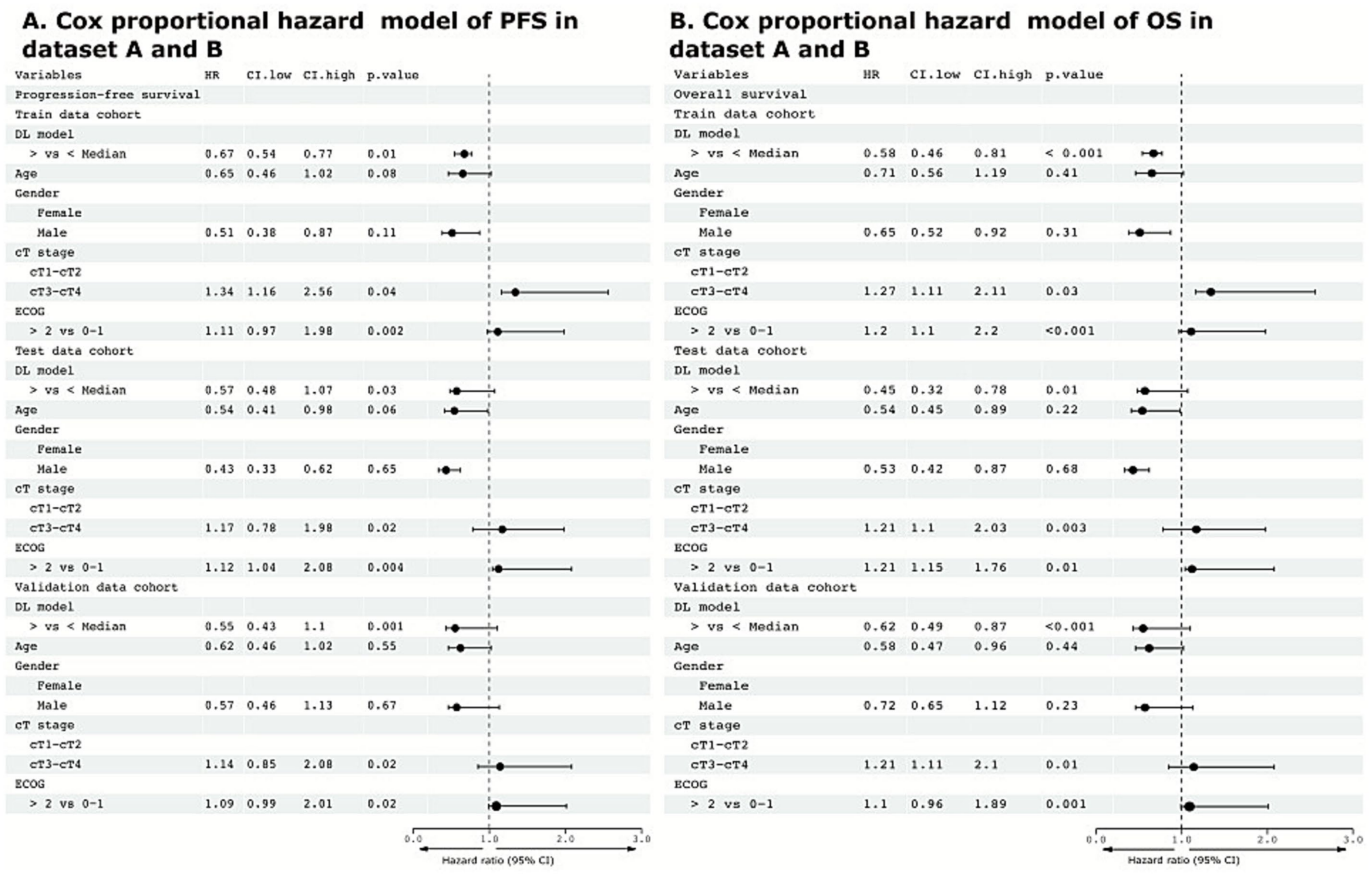
Multivariate analysis in datasets A and B. **(A,B)** Cox proportional hazard model of significant independent predictive factors associated with progression-free survival and overall survival. ECOG, Eastern Cooperative Oncology Group, DL, deep learning; HR, hazard ratio; CI, 95% confidence interval.

To categorize the pathological response, the predictive network was used (Supplementary Figure S3). There was a significant and distinguishable disparity between the responders and gross residual disease. The AUC for responders was 0.785 (*n* = 169, *p* = 0.01, Wilcoxon’s test) and 0.762 for changes in gross tumor volume (*n* = 169, *p* = 0.001, Wilcoxon’s test). We built a combined model of the network to assess the pathologic response to RT and assess the changes in tumor volume (Supplementary Table S4). Again, the model demonstrated strong predictive performance for pathological response (AUC of 0.753, n = 169, *p* = 0.021, Wilcoxon’s test). The prediction probabilities and changes in tumor volume were significantly correlated, achieving a correlation value of 0.61 (*p* = 0.033). Subgroup analysis of other clinical parameters of clinical stage, age, and gender yielded the least significant prediction for pathological response (*p* = 0.631, Wilcoxon’s test). Multivariate and univariate analyses among all covariates demonstrate that DL was an independent predictive factor for both PFS (HR 0.67, 95% CI 0.54–0.77, *p* = 0.01) and OS (HR 0.58, 95% CI 0.46–0.81, *p* < 0.001) (Figures 5A,B, Supplementary Table S5).

### Identification of radiation response through gene expression signature and its biological relevance

**Figure 6:**
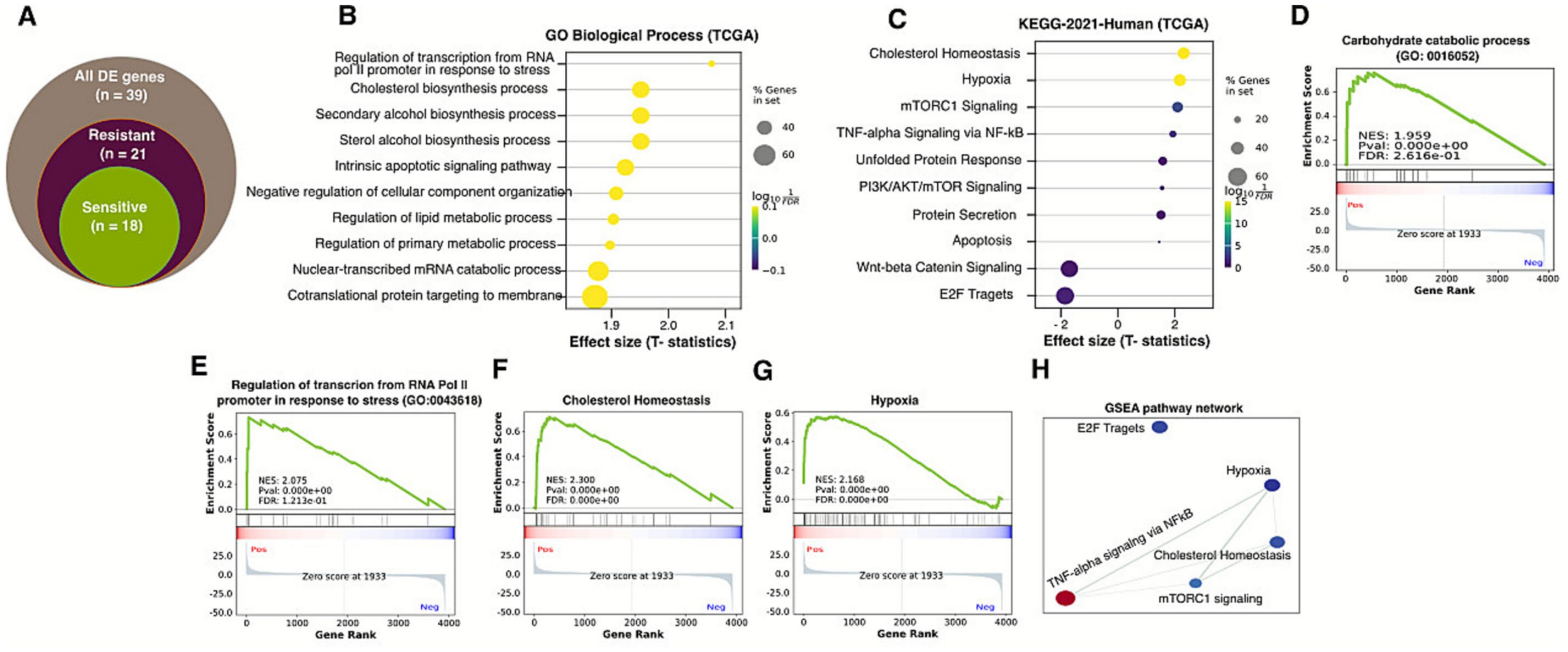
Differential expression analysis from head and neck cancer TCGA dataset. **(A)** Number of differentially expressed genes in resistant and sensitive groups from head and neck cancer TCGA RNA-sequence identified by DESeq2. **(B,C)** Dot plot of GO and KEGG pathways enriched among patients with radiotherapy sensitive and resistant patients. **(D–F)** GSEA analysis of core genes and relation to radio sensitization. GSEA of TPX2, HOXC6, MAP3K, and ADH4. **(H)** Key pathway network identified by GSEA analysis.

To evaluate biomarker contributions in predicting radiation response treatment, we used the TCGA head and neck cancer dataset (*n* = 231) on patients who were treated with radiotherapy only. We have downloaded raw RNA-seq data and patients’ complete clinical metadata, such as age, treatment history, and overall survival and progression-free survival (for details, please refer to the Materials and methods section). To study the gene expression pattern among radiation therapy-resistant and sensitive patients in TCGA gene expression data, the raw expression data were normalized, and 39 (Supplementary Figure S4: Heatmap) differentially expressed genes (DEGs) were identified using DESeq2 following the criteria of Benjamini–Hochberg adjusted *p*-value < 0.05 and logFC > 1 for all samples. The number of differentially expressed genes per group is shown in Figure 6A and Supplementary Figure S2. We then tested the functional enrichment of the DEGs to identify the GO terms associated with radiation resistance and sensitivity using EnrichR and ClusterProfiler. GO and KEGG enrichment analysis showed that DEGs were significantly enriched in co-translational protein targeting to membrane, cholesterol biosynthesis, Wnt-Beta catenin, E2F targets, cholesterol biosynthesis, PI3K/AKT signaling, and hypoxia (Figures 6B,C). Similarly, these DEGs are enriched in carbohydrate catabolic process, regulation of transcription from RNA Pol II promoter in response to stress, cholesterol homoeostasis, and hypoxia (Figures 6D–G).

### Results from the discovery cohort: genes identified with radiation therapy outcome

**Figure 7:**
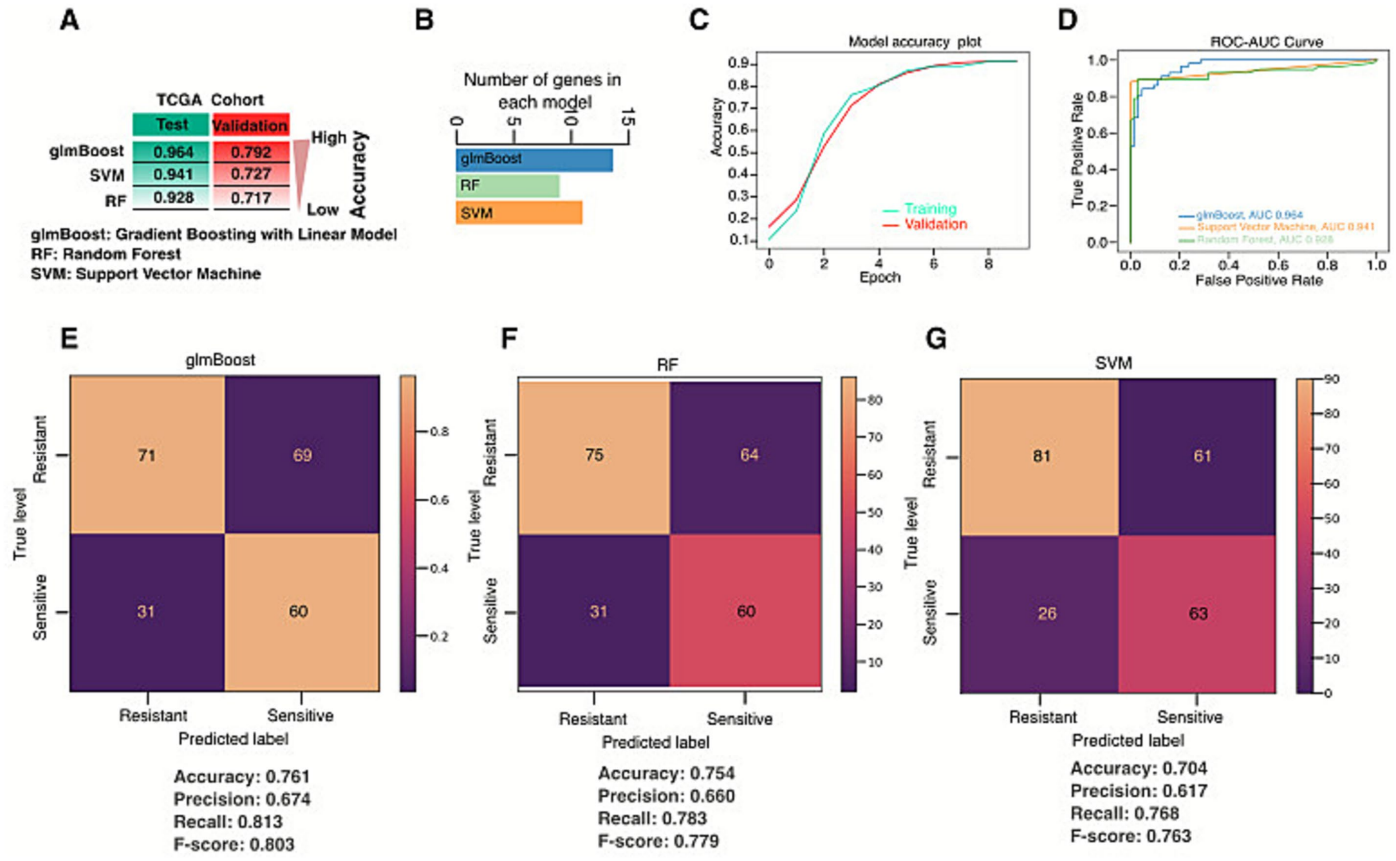
Construction of and validation of models using integrated machine learning. **(A)** Three machine learning algorithms (glmBoost, SVM, and RF), the area under curve (AUC) was calculated for each model for test and validation dataset. **(B)** The number of genes identified by each model. **(C)** AUROC curve for training and validation dataset. **(D)** AUROC curve and values obtained by for the validation cohort using three machine learning algorithms, with optimized hyperparameters. **(E–G)** Confusion matrix obtained by glmBoost, SVM, and RF, respectively, on the validation dataset. The performance metric accuracy, recall (sensitivity), precision, and F1-score are shown below each confusion matrix plot. SVM, support vector machine, RF, random forest.

To accurately predict the outcome of radiation therapy and whether the genes identified in Figure 6 are associated with resistance or not, a feature selection method was employed. Three models, glmBoost (gradient boosting with linear model), RF (random forest), and SVM (support vector machine), were constructed after sample weighting. To identify highly discriminative genes and to distinguish patients with resistant and sensitive groups, we used glmBoost for feature selection and RF for model fitting (Figure 7A). Following the application of three machine learning models, a set of core genes was identified for each feature selection method. The combination of these models identified 13 key genes that are associated with radiotherapy treatment outcome (Supplementary Table S6). The summary AUROC for the treatment prediction model across repeated cross-validation experiments to distinguish patients with resistance from those with sensitivity in the training data set was 0.91 with an F1-score of 0.66. The model generated a sensitivity of 96.3% with a specificity of 83.1%.

In the validation dataset (GSE20020), the model predicted the radiotherapy outcome for the top 7 genes out of 13 genes through the training dataset. The results showed that a group of genes, such as TPX2, HOXC6, MAPK3, KIF14, ESM1, ADH4, and STC2, were consistently captured by these methods, suggesting their potential significance in distinguishing between resistant and sensitive groups (Figure 7B). Each of the genes was selected by the three feature selection models. For the classification of patients with radiation resistance and sensitivity using these 7-core genes, in all cases, models were tuned through CV, StandardScaler, and Extra-Tree Classifier from the RF process using the validation cohort. The hyperparameters and values taken into consideration for each model are assessed. Table 2 shows the accuracy and AUROC values obtained from training and validation datasets via model parameters mentioned above for each model considered. For each model, results are shown with the best set of hyperparameters. In our case, all three models generated identical results, ranging from an accuracy of 0.806–0.813, AUROC ranging from 0.964 to 0.941 in the training dataset. In both datasets, glmBoost performed best among the other two models (Table 3). In the validation cohort, more than 76% of the resistant cases were predicted correctly by all three models (recall), while in the sensitive cases correctly predicted ranged from 61 to 67%. In terms of recall, precision, and F1-score, glmBoost obtained the best results (Table 2). Figures 7C–G show the AUROC and confusion matrix achieved by the three considered ML models for the validation dataset, using as input of the 7-core genes (Supplementary Figure S5).

**Table 2:** Evaluation of machine learning model performance using a 7-gene signature predictive of radiation treatment outcome.

Test type	Sensitivity (recall)	Specificity	AUROC	Accuracy	Precision	F1-score
Validation	0.85	0.68	0.81	0.71	0.63	0.72
Training	0.75	0.65	0.77	0.65	0.71	0.63

**Table 3:** Performance metrics for machine learning (ML) models.

Models	Training cohort (CV)		Validation cohort				
	Accuracy	AUROC	Accuracy	AUROC	Precision	Recall	F1-score
glmBoost	0.813	0.964	0.761	0.865	0.674	0.813	0.803
RF	0.811	0.928	0.754	0.857	0.660	0.783	0.779
SVC	0.806	0.941	0.704	0.851	0.617	0.768	0.763

Performance metrics obtained on the training dataset via CV for the three ML models with optimized hyperparameters, as well as on the validation dataset, using as input the features (genes). glmBoost generated the best results when using as input the features (genes). glmBoost, generalized linear model boost; RF, random forest, SVC, support vector machine; CV, cross-validation; AUROC, area under receiver operating curve.

### Validation of the top seven genes in patient serum samples and proteomics analysis

**Figure 8:**
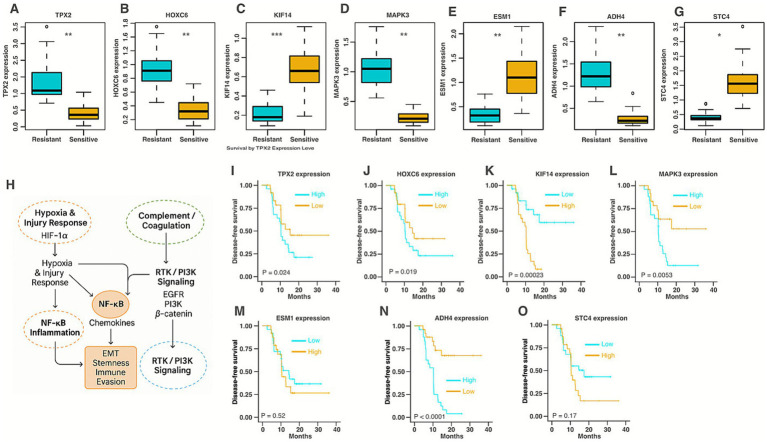
Expression of seven core genes identified by machine learning models. **(A–G)** qRT-PCR mRNA gene expression analysis from resistant (*n* = 25) and sensitive (*n* = 25) laryngeal cancer patients treated with response-adapted RT. **(H)** Graphical summary of networks highlights key mechanistic themes for networks (please see all networks in Supplementary Figure S3). Statistical significance was determined by Student’s *t*-test (*p < 0.05, **p < 0.01). **(I–O)** Kaplan–Meier curves for disease-free survival (DFS) for patients stratified by high and low expression in the signature biomarkers following response-adapted radiation therapy.

Lastly, we have validated 7-core signature genes using patients’ samples from resistant (*n* = 25) and sensitive (*n* = 25) head and neck cancer patients who underwent response-adapted RT. These seven signature genes exhibited significant differential expression between the resistant and sensitive groups (Figures 8A–G). Finally, we have explored protein analysis of patients from the resistant and sensitive groups. Details of the sample processing LC–MS-based protein analysis are described in the materials and methods. The analyses yielded over 350 unique proteins, and 112 proteins showed significant differential expression between resistant and sensitive groups (Supplementary Table S7). The unique protein IDs were converted to gene symbols. Notably, five of our signature genes are also differentially expressed between the two groups, indicative of our model’s prediction accuracy. The five genes were TPX2, ADH4, HOXC6, MAPK3, and KIF14. These results suggest the accuracy and the precision of the model correctly identified the gene features by the models. All 112 DEPs were analyzed using the Ingenuity Pathway Analysis (IPA) to explore the associated biological themes and signaling pathways. Proteomic pathway analysis comparing treatment-sensitive and resistant pharyngeal cancers identified four interconnected signaling networks underlying therapy resistance. Network 1 involved MAPK–JNK–AKT activation with acute-phase and coagulation proteins, reflecting inflammation and survival signaling. Network 3 connected VEGF–ERK–PI3K and complement–NF-κB pathways, indicating angiogenic and immune crosstalk in hypoxic microenvironments. Network 4, centered on NF-κB and transcriptional regulators (SOX2, SP1, EHF), promoted chronic inflammation and stemness. Network 5 integrated HIF-1*α*, EGFR, mTOR, and *β*-catenin signaling, consistent with hypoxia-induced metabolic reprogramming and EMT. Collectively, these pathways form a feedback loop where hypoxia and tissue injury activate complement and coagulation cascades, driving NF-κB– and RTK–PI3K/mTOR–*β*-catenin–mediated inflammation, angiogenesis, and tumor plasticity (Figure 8H, Supplementary Figure S6).

### ML identified signature biomarkers can prognosticate patient outcome

Given the importance of the signature biomarkers in response-adapted RT sensitivity and resistance outcome, we choose two groups of patients (*n* = 50; 25 resistant and 25 sensitive). We extracted RNA from serum samples and used qRT-PCR for relative expression analysis. We observed a significant difference in disease-free survival (DFS) between patients resistant and sensitive patient samples. The DFS for high expression of TPX2, HOXC6, MAPK3, and ADH4 were 11.1, 11.12%, 10.14, and 12.5 and 16.2%, 16.3, 20.3%, respectively (Figures 8I,J,L,N). The overall DFS was reduced among patients with a low expression of KIF14, ESM1, and STC4 signature (Figures 8K,M,O). These findings are consistent with the results obtained in Figure 4.

## Discussion

Management of locally advanced laryngeal cancer remains challenging, with a declining survival rate. The 5-year overall survival (OS) rate for locally advanced larynx cancer remains approximately 50–60%, and a large number of patients require total laryngectomy (Malik et al., 2023; Megwalu and Sikora, 2014). Organ preservation approaches, such as RT, concurrent chemoradiation (CCRT), and induction chemotherapy (ICT) followed by RT or CCRT, can achieve laryngeal preservation in up to 65% of selected patients (Yamakuni et al., 2023; Lagha et al., 2013; Megwalu and Sikora, 2014). Nevertheless, not all locally advanced cases behave uniformly particularly between T3 and T4a stages (Malik et al., 2023; Grover et al., 2015). Although CCRT is widely accepted as a standard treatment approach, the lack of patient selection can adversely affect both OS and survival with functional larynx (SFL).

In clinical practice, monitoring treatment outcomes and evaluating tumors to predict patients’ survival following response-adapted RT plays a critical role in adaptive treatment planning and improving patient outcomes. Currently, radiologists rely on clinical parameters, serial imaging, and changes in tumor load across multiple scans to assess treatment response-an essential component of modern oncology practice. Serial follow-up imaging, in addition to baseline scans, provides valuable insights into treatment efficacy and disease progression. Recent advances in artificial intelligence (AI) and deep learning (DL) have enabled automated, precise evaluation of treatment outcomes beyond traditional visual assessment. DL-based estimation of tumor burden from CT images, combined with prognostic prediction, can aid in evaluating patient outcomes in clinical trials and optimizing response-adapted RT strategies. In this study, we demonstrate that integrating pre-and post-treatment CT scans with gene expression signatures associated with radiation resistance in laryngeal cancer allows for robust outcome prediction using DL and ML-based approaches.

### AI-based deep learning (DL) model performance in assessing radiation treatment outcome from patients’ follow-up CT scans

Quantitative image analysis has yet to become standard in personalizing treatment for head and neck cancer (Hadjiiski et al., 2010; Ling et al., 2025; Zhai et al., 2019). To address the limitations of manual or semi-automated approaches as well as to reduce inter-observer variability, we employed a DL framework combining convolutional neural network (CNNs) and recurrent neural network (RNNs) for time-dependent modeling. Using both pre-and post-treatment CT scans, our model successfully predicted survival, prognosis, and adaptive radiation doses required for local tumor control in patients treated with response-adapted RT for laryngeal cancer. This supports our contention that adaptive RT doses can be more individually tailored when pre- and post-treatment CT scans are used. Remarkably, two key findings emerged from the investigation. One of them is that the model performance improved significantly as the number of post-treatment time points increased. Second, the quantity data used as network input had a substantial impact on the model’s performance. Despite some performance variability, the model maintained consistent AUC values, indicating the reliable prediction of treatment response, the target endpoint, and potential treatment failure.

Although DL approaches have profoundly enhanced clinical diagnosis from CT images, stratifying patients into high- and low-risk groups remains challenging. Previous studies have developed and validated DL models for risk stratification based on radiographic data (Bang et al., 2023; Le et al., 2022), most focused on patient outcomes rather than organ preservation or locoregional failure. Our neural network approach effectively distinguishes between high-and low-mortality risk groups, showing significant differences in overall survival. Furthermore, the model accurately identified risks of local recurrence when two post-treatment follow-up scans (obtained 2 months after response-adapted RT) were provided as input. The other two outcomes, progression-free survival and locoregional recurrence, also reliably stratified.

For model validation and response prediction, we included post-RT and pre-surgical follow-up time points in dataset B (primary RT). Despite being completely blinded throughout model development, the model successfully distinguished pathological responders from patients with residual disease in the initial RT cohort. Predictions correlated strongly with primary tumor size response, confirming that volumetric tumor changes reflect treatment efficacy. Notably, the inclusion of multiple follow-up time points enabled real-time assessment of dynamic tumor changes, made possible through RNNs that can process incomplete longitudinal data an essential feature for retrospective studies like ours.

### Machine learning (ML) approaches to identify signature biomarkers from gene expression data associated with radiation therapy

In this segment of our results, we present data demonstrating the gene expression signature by developing an integrative approach to establish a consistent HNSCC radiotherapy treatment outcome signature using samples from publicly available datasets. Using an ML framework, we identified 13 genes consistently associated with radiotherapy treatment response through repeated leave-one-out cross-validation. Furthermore, validation identified seven core genes that formed a fixed classifier reliably distinguishing radiotherapy sensitivity from radiotherapy-resistant cases. Among the tested models, the glmBoost algorithm exhibited the highest performance and reproducibility compared to the random forest (RF) and support vector machine classifier (SVM) models.

Several of the identified genes, including TPX2, HOXC6, KIF14, MAPK3, ESM1, ADH2, and STC2, have established roles in chemoradiotherapy (Zhu et al., 2024; Moon et al., 2012; Matic et al., 2023; Ngan et al., 2022; Xu et al., 2019; Al-Shamma et al., 2023; Qie and Sang, 2022). TPX2 plays a critical role in various cellular and molecular processes resulting in growth and metastasis, mitotic survival, and is linked to the radiosensitivity of tumor cells (Huang et al., 2014; Kim et al., 2023). HOXC6 is involved in cell proliferation, survival, and metastasis as well as many cellular processes (Li et al., 2022). Furthermore, deregulated functions of HOXC6 may directly influence cellular sensitivity and efficacy of radiation and chemotherapy. High expression of HOXC6 is associated with poor prognosis, higher risk of death, and is associated with immune-modulatory genes (Du et al., 2014; Zhang et al., 2013). As a microtubule-based motor protein, KIF14 is involved in growth and cell motility in tumor cells and is associated with poor clinical outcome (Corson and Gallie, 2006), which causes the response to radiation therapy. Endothelial cell-specific molecule 1 (ESM1) is an important proto-oncogene that may influence chemo and radio sensitivity by regulating PI3K/AKT signaling pathway, and ESM1 can be activated by inflammation and cytokines as well as play a role in angiogenesis (Xu et al., 2019). Alcohol dehydrogenase-4 (ADH4) plays key role in radiation resistance, inhibit the DNA damage and cell death induced by radiotherapy, and ADH4 has been identified as a cancer stem marker in different cancer (Januchowski et al., 2013; Al-Shamma et al., 2023) Stanniocalcin 2 (STC2) overexpressed in many cancer including head and neck cancer is correlated with tumor development and metastasis and play substantial role in radiation sensitivity via activating PI3K/AKT/and Snail signaling (Qie and Sang, 2022; Yang et al., 2016). Collectively, these evidences confirm that our core gene signature is biologically relevant and reliable for predicting radiosensitivity in laryngeal cancer. Importantly, our gene expression classifier demonstrated prognostic relevance in predicting radiotherapy outcome. This ML-driven approach enables the identification of gene signatures associated with treatment response, optimizing individualized therapy. However, our findings from gene expression signature may limit direct prediction of survival outcome due to the involvement that other patient-specific confounding factors. Overall, the novelty of this study lies in integrating patients’ follow-up images with gene expression-based ML models to identify patients most likely to respond to response-adaptive RT.

## Limitations

This study has several limitations that should be acknowledged. The first relates to the relatively small sample size and single-center of the study. Expanding the dataset and enrolling more centres, and employing more CNNs, may substantially enhance the prediction accuracy and help address this issue. Because the study is retrospective, the influence of confounding variables and potential bias cannot be completely excluded. Although three-dimensional imaging could more accurately represent tumor biology and potentially strengthen model performance, this work utilized two-dimensional images. Incorporating 3D imaging in future research may yield a more comprehensive understanding of tumor characteristics. DL and ML models often operate as “black boxes,” where the internal reasoning behind predictions remains difficult to interpret despite their strong performance. Our survival analysis relied solely on CT image features, excluding clinical factors such as age, sex, histology, or smoking history, which may also hold prognostic values. Integration of RNA-seq and microarray datasets may have introduced variability linked to shared gene signatures associated with radiotherapy resistance. Furthermore, while CT scans can be obtained at multiple time points during a patient’s follow-up, gene expression profiles reflect only a single snapshot, which might influence the interpretation of results. Future studies that include longitudinal gene expression analysis across multiple treatment stages could provide deeper insight into the temporal dynamics of radiotherapy response and outcome. Finally, because deep learning models we used in image analysis may not be much informative on the biological difference. Therefore, we separately used ML models, such as RF, SVC, and glmBoost ML learning approach to identify genes or biomarkers. Lastly, when building DL model we have overlooked for image analysis, like visualizing GRAD-CAM generated features which influence the model’s decision and to verify the model on focusing anatomically relevant region, identify potential biases in learning pattern and to improve model performance. Future studies will be design to build the trustworthy of AI system to integrate the diagnostic workflow. Despite all these constraints, our DL effectively predicted survival and radiotherapy outcome, while the ML models identified treatment response based on signature genes. Expanding cohort size and refining models’ architecture in subsequent work should further enhance the robustness and vulnerability of the predictive models.

In conclusion, our integrated DL and ML models demonstrate the potential to predict tumor phenotype tracking, pathological response in pre- and post-RT, and outcome of radiotherapy sensitivity in laryngeal carcinoma using serial CT scans and gene expression data. These approaches could provide a reliable, noninvasive approach, which may have potential clinical implications for adaptive and precision therapy.

## Data Availability

The original contributions presented in the study are included in the article/Supplementary material, further inquiries can be directed to the corresponding author.
