# Supplementary material for: Deep learning and machine learning integration of radiomics and transcriptomics predicts response-adapted radiotherapy outcome and radiosensitivity in resectable locally advanced laryngeal carcinoma
Source: Front Artif Intell. 2026 Jan 12;8:1738174. doi: 10.3389/frai.2025.1738174 (PMC12832843; doi:10.3389/frai.2025.1738174)
Supplement: Supplementary file 1 [file Data_Sheet_1.PDF]

**Deep Learning and Machine Learning Integration of Radiomics and Transcriptomics Predicts Response-Adapted Radiotherapy Outcome and Radiosensitivity in Resectable Locally Advanced Laryngeal Carcinoma**

*Shafatujjahan et al.*

Corresponding author: sislam83@kfshrc.edu.sa

This PDF file includes Supplementary Figures S1 to S6
Supplementary Tables S1 to S6. Supplementary Table S7 in .xlsx format attached separately

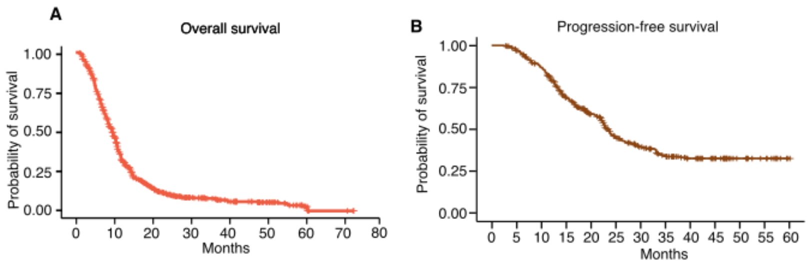


**Supplementary Figure S1.** Kaplan-Meier for A) Overall survival and B) progression-free survival of all patients.

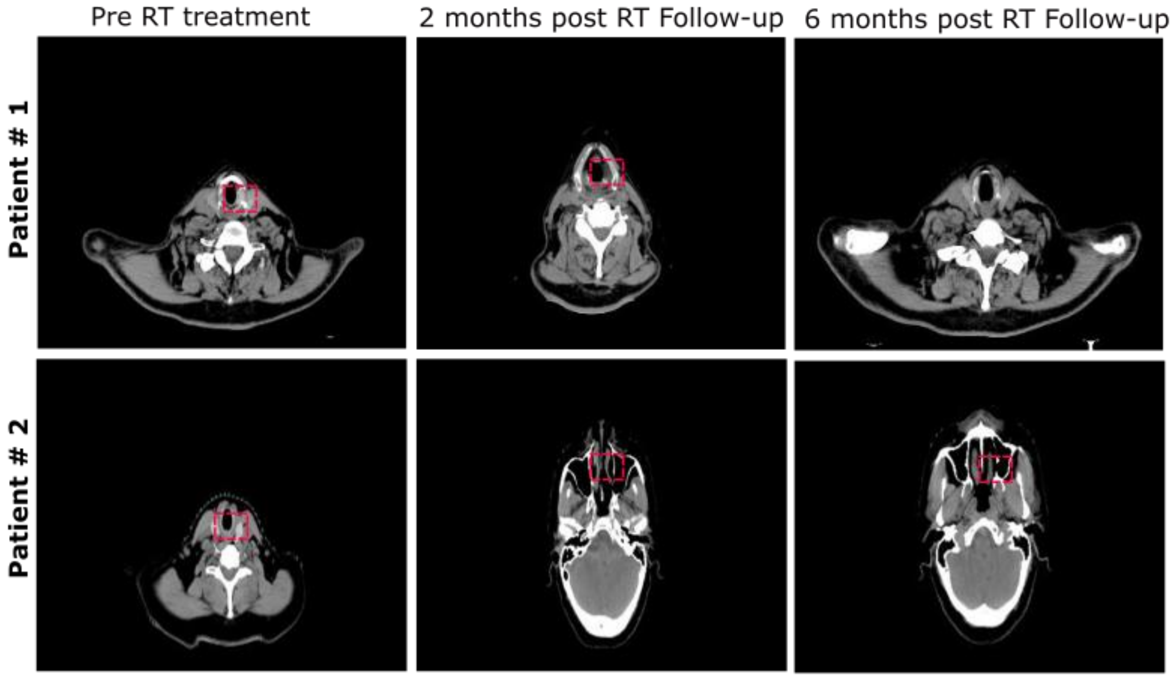


**Supplementary Figure S2: Serial patient CT scans from laryngeal cancer patients.** Serial CT scans were taken of two representative patients with stage IV before response-adapted RT treatment and 2 and 6 months after response-adapted RT treatment. A square, dotted, red-colored line identifies the single seed point input into the neural networks.

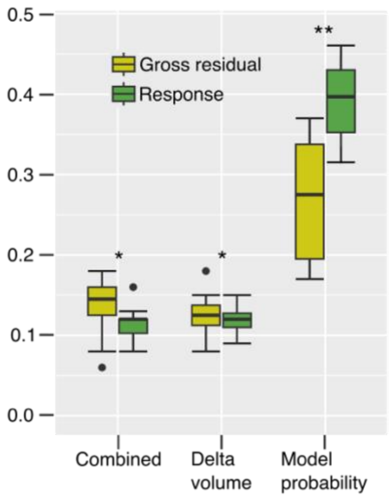


**Supplementary Figure S3.** Model probability and changes in tumor volume after radiation therapy. Comparison of prediction was assessed in changes of tumor volume and two parameters. Wilcoxon test $p < 0.05$.

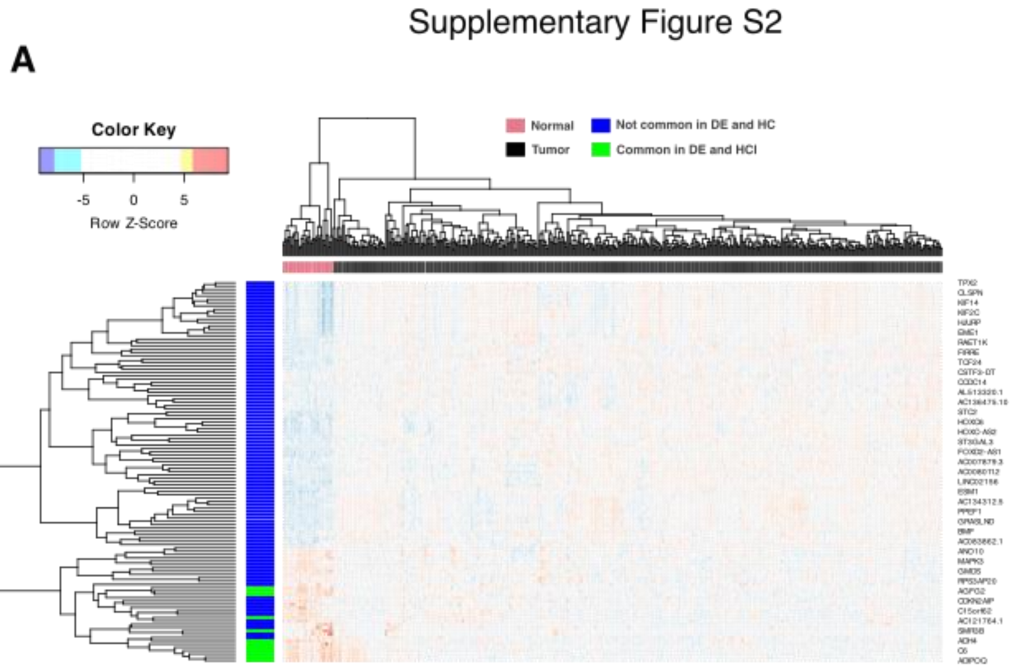


**Supplementary Figure S4. Differential gene expression analysis from head and neck cancer TCGA dataset.** Heatmap showing the differentially expressed genes (DEGs) among patients with radiotherapy-resistant and sensitive patients in the training dataset (TCGA head and neck cancer RNA-sequence raw data).

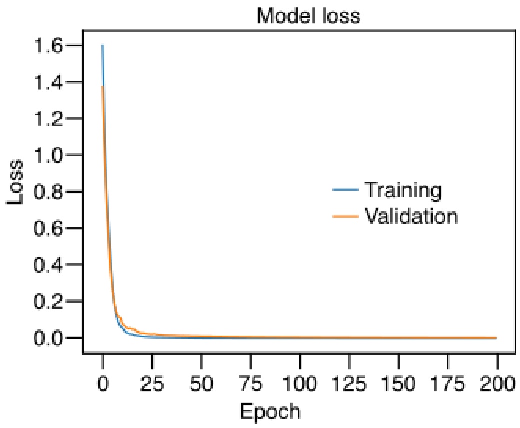


**Supplementary Figure S5.** The loss of function in training dataset.

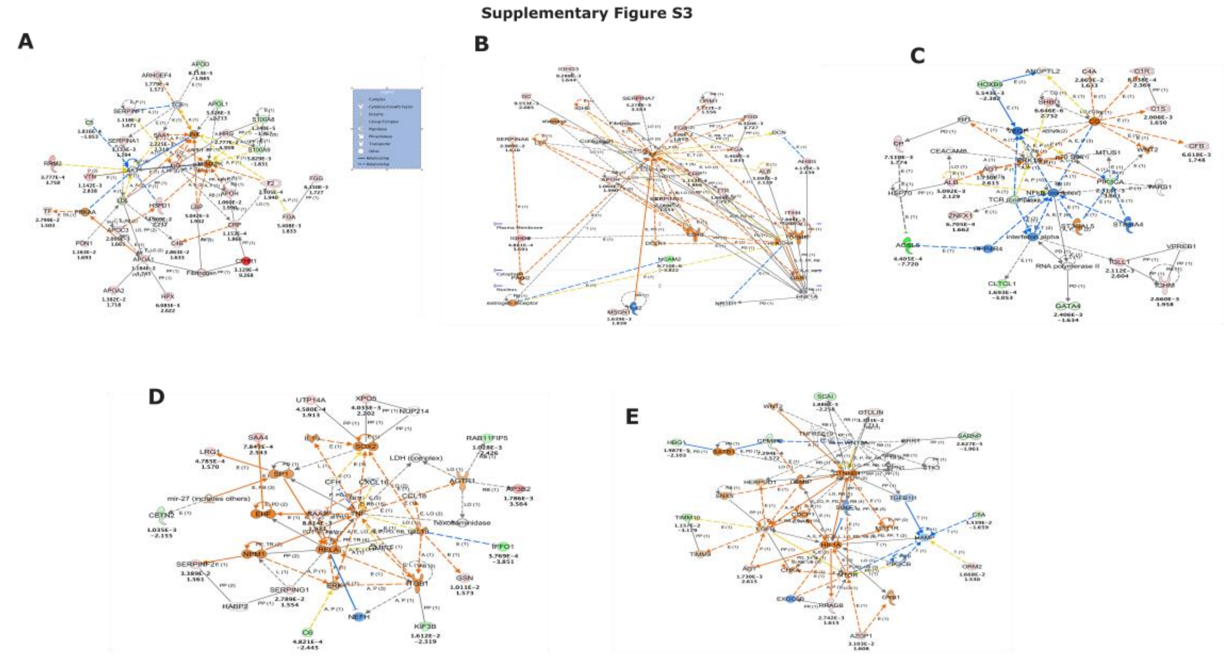


**Supplementary Figure S6. Integrated mechanistic network of response-adapted RT therapy resistance in laryngeal carcinoma. A-E** summarizes the signaling networks identified from differentially expressed proteins between treatment resistant and sensitive patients' serum samples.

| | Supplementary table S1 | |
|---|---|---|
| Genes | Forward | Reverse |
| TPX2 | GAGGGCCTTTCTGGTTCTCT | CTCCTGTAGTCTGGCCTCCT |
| HOXC6 | ACCGACCAGGTAAAGGCAAA | GGGGAAAAGGGCCTGTAGAC |
| KIF14 | TGGCATTAAGAGTTTAGCAGATACT | AGGGGTAAGGGGCATGTCT |
| MAP3K | GAATGTAGCCCGCGAGAGAA | GGAGTTGCCAGGAGAAGGAC |
| ESM1 | GCAAAGACCACGACTGGAGA | GCAAAGACCACGACTGGAGA |
| ADH4 | CACCAAGGGCAAAGTTATTAAATGC | CAGCCCCATAGCCAGTTGAA |
| STC4 | GGCGCTGGGTAACCTCTATC | CCAGCGTTGACCAAACAGTG |

Supplementary Table S2: Baseline patient characteristics of the Training and Testing cohort of dataset A (Response-adapted radiation treatment group [RART]. Median (range) was reported for continuous variables and counts (percentage) for categorical variables.

| Variables | N = 476[1] | RART[2]: Training cohort N = 318[1] | RART[2]: Testing cohort N = 158[1] | p-value[3] | q-value[4] |
|---|---|---|---|---|---|
| **Sex** | | | | | |
| Male | 374 (79%) | 247 (78%) | 127 (80%) | 0.5 | 0.7 |
| Female | 102 (21%) | 71 (22%) | 31 (20%) | | |
| **Age, median (range), y** | 62.12 (9.8) | 61.0 (8.9) | 61.5 (10.1) | 0.2 | 0.4 |
| **Subsites** | | | | | |
| Epiglottis | 131 (28%) | 98 (31%) | 3 (2%) | 0.012 | 0.027 |
| Glottis | 76 (16%) | 49 (15%) | 27 (17%) | | |
| Subglottis | 80 (17%) | 61 (19%) | 19 (12%) | | |
| Supraglottis | 72 (15%) | 33 (10%) | 39 (25%) | | |
| Vocal cord | 117 (25%) | 77 (24%) | 70 (44%) | | |
| **CCI-score** | | | | | |
| 0 | 194 (41%) | 140 (44%) | 54 (34%) | 0.8 | 0.8 |
| 1 | 171 (36%) | 124 (39%( | 47 (30%) | | |
| 2 | 70 (15%) | 37 (12%) | 33 (21%) | | |
| >3 | 41 (9%) | 17 (5%) | 24 (15%) | | |
| **Clinical T-stage** | | | | | |
| T1 | 22 (5%) | 13 (4%) | 9 (6%) | 0.7 | 0.8 |
| T2 | 43 (9%) | 21 (7%) | 22 (14%) | | |
| T3 | 197 (41%) | 133 (42%) | 64 (40%) | | |
| T4 | 214 (45%) | 151 (48%) | 63 (40%) | | |
| **Clinical N-stage** | | | | | |
| N0 | 17 (4%) | 11 (3%) | 6 )4%) | < 0.0001 | < 0.001 |
| N1 | 19 (4%) | 9 (3%) | 10 (6%) | | |
| N2 | 310 (65%) | 201 (63%) | 109 (69%) | | |
| N3 | 130 (27%) | 97 (30%) | 33 (21%) | | |
| **Clinical stage** | | | | | |
| III | 64 (13%) | 55 (17%) | 9 (6%) | 0.8 | 0.8 |
| IVA | 329 (69%) | 198 (62%) | 131 (83%) | | |
| IVB | 83 (17%) | 65 (20%) | 18 (11%) | | |
| **Concurrent chemotherapy** | 146 (31%) | 129 (41%) | 17 (11%) | 0.007 | 0.019 |
| **Radiation techniques** | | | | | |
| 3DCRT | 18 (4%) | 15 (5%) | 3 (2%) | 0.006 | 0.019 |
| IMRT | 450 (95%) | 298 (94%) | 152 (96%) | | |
| VMAT | 8 (2%) | 5 (2%) | 3 (2%) | | |
| **ECOG** | | | | | |
| 0 | 14 (3%) | 11 (3%) | 3 (2%) | < 0.001 | < 0.001 |
| 1 | 460 (96%) | 306 (96%) | 154 (97%) | | |
| >2 | 2 (1%) | 1 (1%) | 1 (1%) | | |
| **Pretreatment evaluation** | | | | | |
| Laryngeal preservation | 142 (30%) | 109 (34%) | 33 (21%) | 0.5 | 0.7 |
| Total laryngectomy | 334 (70%) | 209 (66%) | 125 (79%) | | |

[1] Mean (SD); n (%)
[2] RART- Response-adapted radiotherapy
[3] Wilcoxon rank sum test: Pearson's Chi-squared test: Fishers exact test
[4] False discovery rate correction for multiple testing

| Supplementary Table S3: AUC values for the pre-treatment images and clinical models only images with regard to prediction of overall survival, progression-free survival, locoregional-free survival, and survival with a functional larynx | | | | |
|---|---|---|---|---|
| | **Pre-treatment (n = 158)** | | **Clinical (n = 158)** | |
| **5-year** | **AUC** | **Rank-sums** | **AUC** | **Rank-sums** |
| **Progression-free survival** | 0.782 | 0.272 | 0.801 | 0.538 |
| **Overall survival** | 0.746 | 0.183 | 0.773 | 0.374 |
| **Locoregional-free survival** | 0.771 | 0.243 | 0.816 | 0.651 |

| Supplementary Table S4: AUC values for the validation dataset (Dataset B; radical RT) therapy only using two input models trained on the training dataset, with regard to the prediction of survival, progression-free survival, locoregional-free survival | | |
|---|---|---|
| 5- year | Area Under Curve (AUC) | Rank-sums |
| Progression-free survival | 0.751 | 0.032 |
| Overall survival | 0.802 | 0.192 |
| Locoregional-free survival | 0.773 | 0.063 |

| Supplementary Table S5: Univariate results identify variables affecting progression-free survival and overall survival to treatment. | | | | |
|---|---|---|---|---|
| | **Disease-free survival** | | **Overall survival** | |
| **Variables** | **HR (95 CI)** | ***P* value** | **HR (95% CI)** | ***P* value** |
| *Training set (dataset A)* | | | | |
| DL model | | | | |
| > vs ≤ Median | 0.54 (0.36-0.85) | **0.020** | 0.53 (0.46-0.78) | **0.031** |
| Age | 0.59 (0.41-1.02) | 0.225 | 0.67 (0.56-1.05) | 0.246 |
| *Gender* | | | | |
| Female | | | | |
| Male | 0.56 (0.43-0.92) | 0.19 | 0.46 (0.35-0.97) | 0.89 |
| *cT stage* | | | | |
| cT1-cT2 | | | | |
| cT3-cT4 | 1.23 (0.65-2.21) | 0**.05** | 1.15 (1.02-2.21) | **0.05** |
| *ECOG* | | | | |
| ≥ 2 vs 0-1 | 1.16 (0.87-2.03) | **0.04** | 1.03 (0.87-2.04) | **0.02** |
| *Test data set (dataset A)* | | | | |
| DL model | | | | |
| > vs ≤ Median | 0.65 (0.44-1.03 | **< 0.001** | 0.61 (0.55-1.01) | **0.01** |
| Age | 0.48 (0.35-0.97) | 0.11 | 0.53 (0.47-0.99) | 0.08 |
| *Gender* | | | | |
| Female | | | | |
| Male | 0.58 (0.48-1.08) | 0.76 | 0.48 (0.41-0.87) | 0.66 |
| *cT stage* | | | | |
| cT1-cT2 | | | | |
| cT3-cT4 | 1.24 (1.06-2.26) | **0.03** | 1.12 (1.05-2.05) | **0.04** |
| *ECOG* | | | | |
| ≥ 2 vs 0-1 | 1.06 (0.98-1.95) | **0.03** | 1.08 (0.89-2.01) | **0.03** |

| | | | | |
|---|---|---|---|---|
| *Validation dataset (dataset B)* | | | | |
| DL model | | | | |
| > vs ≤ Median | 0.62 (0.48-0.87) | **0.004** | 0.62 (0.57-0.89) | **0.02** |
| Age | 0.58(0.47-1.12) | 0.82 | 0.49 (0.41-0.96) | 0.98 |
| *Gender* | | | | |
| Female | | | | |
| Male | 0.46 (0.37-0.89) | 0.23 | 0.66 (0.55-1.01) | 0.75 |
| *cT stage* | | | | |
| cT1-cT2 | | | | |
| cT3-cT4 | 1.12 (1.02-2.26) | **0.01** | 1.05 (0.92-1.16) | **0.01** |
| *ECOG* | | | | |
| ≥ 2 vs 0-1 | 1.09 (.87-2.16 | **0.02** | 1.1 (0.95-1.87) | **0.01** |
| HR, hazard ratio; CI, Confidence interval; ECOG, Eastern Cooperative Oncological Group; DL, Deep learning | | | | |

Supplementary Table S6: Gene feature identified through machine learning predictive of radiotherapy outcome the training dataset (TCGA head and neck cancer) strength of association analyzed via repeated cross-validation.

| Sl. No | Gene symbol | Gene ID | CV Fraction | Gene description |
|---|---|---|---|---|
| 1 | TPX2 | ENSG00000088325.17 | 1.00 | TPX2 microtubule nucleation factor |
| 2 | KIF14 | ENSG00000118193.13 | 1.00 | Kinesin family member 14 |
| 3 | ESM1 | ENSG00000164283.13 | 1.00 | Endothelial cell specific molecule 1 |
| 4 | STC2 | ENSG00000113739.11 | 0.993 | Stanniocalcin 2 |
| 5 | HOXC6 | ENSG00000197757.8 | 0.975 | Homeobox C6 |
| 6 | ADH4 | ENSG00000198099.10 | 0.934 | Alcohol dehydrogenase 4 |
| 7 | MAPK3 | ENSG00000102882.13 | 0.911 | Mitogen-activated protein kinase 3 |
| 8 | RAET1K | ENSG00000218358.4 | 0.901 | Retinoic acid early transcript 1K |
| 9 | TCF24 | ENSG00000261787.3 | 0.896 | Transcription factor 24 |
| 10 | BMF | ENSG00000104081.15 | 0.857 | Bcl2 modifying factor |
| 11 | GMDS | ENSG00000112699.12 | 0.841 | GDP-mannose 4,6-dehydratase |
| 12 | EME1 | ENSG00000154920.16 | 0.838 | Essential meiotic structure-specific endonuclease 1 |
| 13 | SMR3B | ENSG00000171201.12 | 0.814 | Submaxillary gland androgen regulated protein 3B |
